# Co-fertilization of Sulfur and Struvite-Phosphorus in a Slow-Release Fertilizer Improves Soybean Cultivation

**DOI:** 10.3389/fpls.2022.861574

**Published:** 2022-05-10

**Authors:** Stella F. Valle, Amanda S. Giroto, Gelton G. F. Guimarães, Kerstin A. Nagel, Anna Galinski, Jens Cohnen, Nicolai D. Jablonowski, Caue Ribeiro

**Affiliations:** ^1^Department of Chemistry, Federal University of São Carlos, São Carlos, Brazil; ^2^Embrapa Instrumentation, São Carlos, Brazil; ^3^Agricultural Research and Rural Extension Company of Santa Catarina, Itajaí, Brazil; ^4^Institute of Bio- and Geosciences, IBG-2: Plant Sciences, Forschungszentrum Jülich GmbH, Jülich, Germany

**Keywords:** struvite, sulfur, polysulfide, soybean, root, fertilizer, rhizotron

## Abstract

In face of the alarming world population growth predictions and its threat to food security, the development of sustainable fertilizer alternatives is urgent. Moreover, fertilizer performance should be assessed not only in terms of yield but also in root system development, as it impacts soil fertility and crop productivity. Fertilizers containing a polysulfide matrix (PS) with dispersed struvite (St) were studied for S and P nutrition due to their controlled-release behavior. Soybean cultivation in a closed system with St/PS composites provided superior biomass compared to a reference of triple superphosphate (TSP) with ammonium sulfate (AS), with up to 3 and 10 times higher mass of shoots and roots, respectively. Root system architectural changes may explain these results, with a higher proliferation of second order lateral roots in response to struvite ongoing P delivery. The total root length was between 1,942 and 4,291 cm for plants under St/PS composites and only 982 cm with TSP/AS. While phosphorus uptake efficiency was similar in all fertilized treatments (11–14%), St/PS achieved a 22% sulfur uptake efficiency against only 8% from TSP/AS. Overall, the composites showed great potential as efficient slow-release fertilizers for enhanced soybean productivity.

## Graphical Abstract

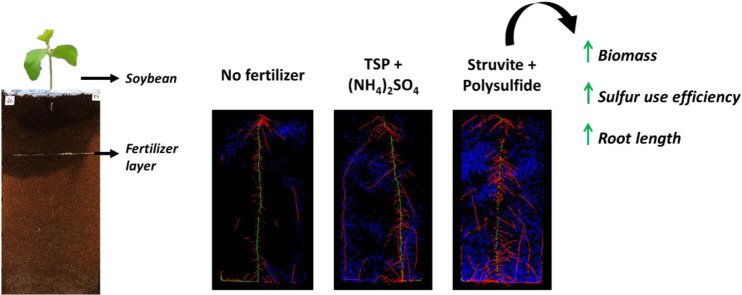

## Introduction

Phosphorus (P) is vital for plant nutrition and growth, and one of the most limiting elements for crop production. Agriculture represents nearly 90% of P use worldwide, yet, its current consumption rate has been unsustainable and incompatible with the natural cycle of the element, as phosphate rocks are non-renewable resources (Cordell et al., 2009; Scholz et al., 2013; Chowdhury et al., 2017). Moreover, the efficiency of P fertilizers is significantly restricted by soil immobilization processes of sorption and precipitation (Rech et al., 2018). Conventional P fertilizers are readily soluble and thus release P faster than plants can uptake, contributing to soil fixation. These sources are also highly susceptible to runoff losses, causing eutrophication of water bodies and associated environmental damages (Chien et al., 2011; International Plant Nutrition Institute [IPNI], 2019).

Therefore, sustainable solutions for phosphorus fertilization are an urgent concern facing food security. Struvite (MgNH_4_PO_4_⋅6H_2_O) is a promising alternative, recovered from municipal wastewater streams, which could reduce the P cycle gap (Rahman et al., 2014; Kataki et al., 2016; Talboys et al., 2016; Yetilmezsoy et al., 2017; Mehta et al., 2018; Rech et al., 2018). In addition, it serves as a source of nitrogen (N) and magnesium (Mg), essential macronutrients for plant development (Rahman et al., 2014; Kataki et al., 2016). Moreover, struvite is considered a slow-release fertilizer due to its low water solubility, which leads to reduced losses and a prolonged residual value to crops (Talboys et al., 2016). Nevertheless, low solubility may also result in an inadequate phosphorous supply to plants. Struvite dissolution can be significantly improved in acidic conditions and is highly affected by particle size, and it is solubilized at a much slower rate when in granular form than as a powder (Degryse et al., 2017; Tansel and Monje, 2018; Robles-Aguilar et al., 2019; Hertzberger et al., 2020). For field application, however, fertilizers are usually managed as granules or pellets, which are easier for handling and storage (Giroto et al., 2020).

Therefore, by controlling local acidity and particle size, struvite can provide P fertilization more efficiently and safely. Recently, our research group accomplished both of these criteria with the development of fertilizer composites based on a polysulfide matrix containing dispersed ground struvite (Valle et al., 2021). Matrices are strategic for getting around the particle size problem, as they can be processed as granules, while simultaneously keeping small P particles from agglomerating (Ribeiro and Carmo, 2019). At the same time, the matrix acts as a barrier, preventing a fast P delivery (Mann et al., 2019). The studied polysulfide is an especially interesting material as it can provide sulfur to plants, an important macronutrient for plant growth that is frequently unavailable in agricultural soils (Scherer, 2001; Lucheta and Lambais, 2012; Valle et al., 2019). The polysulfide structure contains polymeric sulfur chains, obtained by inverse vulcanization of elemental sulfur (S_8_), a residue from the oil industry (Chung et al., 2013; Abbasi et al., 2019; Chalker et al., 2019; Zhang et al., 2019; Park and Leitao, 2021). For plant uptake, both the polysulfide and pure S_8_ have to be oxidized in the soil to sulfate, a slow rate process promoted by soil microorganisms (Germida and Janzen, 1993; Degryse et al., 2016). The polysulfides from our previous studies displayed superior oxidation compared to S_8_, especially when combined with struvite (Valle et al., 2019, 2021). Additionally, sulfate formation lowered the local pH, assisting struvite dissolution (Valle et al., 2021).

Despite its potential as an environmentally friendly fertilizer, the struvite-polysulfide effects on plants are still unknown, and its dynamics in a soil-plant system should be further investigated. Most importantly, we were interested in understanding the fertilizer’s influence on root development and spatial distribution of roots in the growth medium, as an indication of how the fertilizer can be accessed by plants. In the current work, we investigated the effect of struvite-polysulfide fertilizers on nutrient uptake, biomass formation, and root system architecture. Soybean (*Glycine max* L.) was selected for the study, as a plant with high protein content and high S demand (Zhao et al., 2008; Ibañez et al., 2020). We hypothesized that soybean would respond differently to the struvite-polysulfide composites compared to a soluble reference, due to the controlled delivery of P. In addition, we hypothesized that the S chemical structure from the fertilizers would affect S supply and soybean root system traits, as polysulfides need to be biologically converted to sulfate.

## Materials and Methods

### Preparation of Composites

**FIGURE 1:**
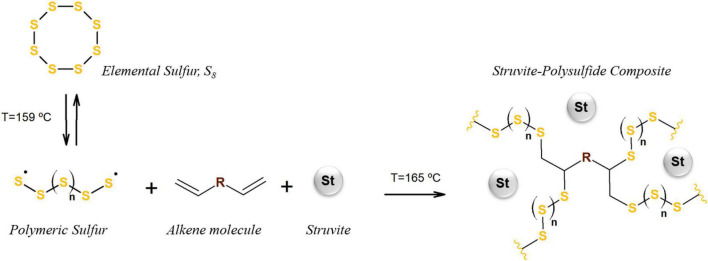
Preparation of the struvite–polysulfide fertilizer composite (generic structure). Elemental sulfur undergoes ring-opening polymerization and reacts with alkene molecules (in this work, soybean oil), in the presence of ground struvite, producing the polysulfide matrix with dispersed phosphate particles.

Composite fertilizers containing a polysulfide matrix and dispersed struvite particles were prepared as described by Valle et al. (2021), illustrated in Figure 1. Prior to the preparation of the composites, struvite (Ostara Crystal Green, United Kingdom) was pulverized in an orbital mill (Servitech CT 241, Tubarão, Brazil) with alumina balls, followed by sieving (<0.15 mm). The polysulfide structure was obtained using the inverse vulcanization between elemental sulfur (S_8_; Synth, Brazil) and soybean oil (Liza, Brazil), each at 50 wt%. This method is solvent-free and has no byproduct formation. The reaction was conducted in the presence of ground struvite, with different mass ratios (25, 50, and 75 wt% of struvite in relation to the composite). All compounds were mixed in a flask and the system was kept under constant agitation and heat, using a mechanical stirrer and oil bath. The temperature was kept at approximately 165°C, allowing the ring-opening polymerization (ROP) of S_8_, followed by the reaction between bi-radical polymeric sulfur chains and unsaturated bonds from soybean oil, until a light brown material was obtained. In order to compare the polysulfide-based composites with the struvite reference (1 mm granules) in the greenhouse experiment, the composites were roughly ground (<1 mm) in a blade grinder (Philco, Brazil).

### Greenhouse Experiment

To test the agronomic efficiency of the St/PS composite fertilizers and their effect on root and shoot soybean plant performance, an experiment was conducted under controlled greenhouse conditions at the Institute of Bio- and Geosciences, IBG-2: Plant Sciences, *Forschungszentrum Jülich GmbH*, Germany (50°54′36′′N, 6°24′49′′E), from May to July 2020. An average temperature of 23°C and air humidity of 48% was maintained at the greenhouse over this period.

In order to evaluate the combined effect of struvite and polysulfide, the following treatments were applied: no fertilizer (control); a positive reference with the highly soluble sources triple superphosphate for P and ammonium sulfate for S (TSP/AS); mixed pure struvite and elemental sulfur powder (St/S8); and ground fertilizer composites with different mass ratios of struvite and polysulfide–St 25/PS, St 50/PS, and St 75/PS (respectively, with 25, 50, and 75 wt% of struvite). A fixed ratio of 50 mg of S per kg of soil was established for all fertilized treatments. To achieve a P concentration of 200 mg per kg of soil, additional struvite was supplied with the composite treatments. Since the aim was to study P and S effects on the plant development, a fixed dose of N was supplied to all fertilized treatments to make sure it was sufficiently provided and not a limiting factor to soybean growth. Nitrogen was supplemented with ammonium nitrate in all fertilized treatments to complete 300 mg of N/kg of soil. Potassium, zinc, and copper were also supplemented in concentrations of 200, 5, and 1.5 mg/kg, respectively, using a nutrient solution containing KCl, ZnCl, and CuSO_4_. Detailed information on nutrient content and supply can be found in Supplementary Table 1.

**FIGURE 2:**
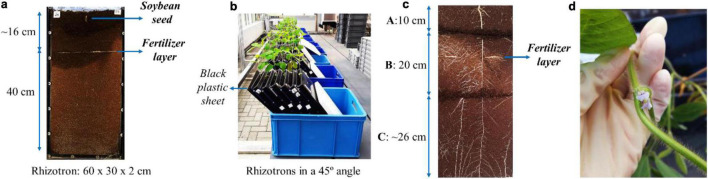
**(a)** Rhizotron with a fixed layer of fertilizer and pre-germinated soybean seedling; **(b)** Rhizotrons during cultivation; **(c)** Substrate and root sampling in layers A (top layer, 10 cm), B (middle layer, 20 cm, including the fertilizer layer), and C (bottom layer, ∼26 cm); **(d)** Flower bloom 30 days after sowing.

Peat substrate (“Nullerde,” Einheitserde/Patzer Erden, Germany) was selected as a growth medium due to an assumed high microbial activity in organic-rich environments, which is necessary to promote S oxidation from the polysulfide and S_8_. The substrate consisted of a mixture of 30% clay and 70% white peat, with no prior addition of fertilizers. Detailed substrate characterization can be seen in Supplementary Table 2. Before the experiment, the substrate was shredded and sieved (<0.7 cm) to remove coarse particles. Flat rhizotrons (60 cm × 30 cm × 2 cm) (Nagel et al., 2012) were filled with 2 kg of the substrate (approximately 3.36 dm^3^), with 10 replicates per treatment. Fertilizers were added 8 days before sowing, placed on a fixed layer at 40 cm from the bottom of the rhizotron (at approximately 16 cm from the substrate surface, 20 cm from the rhizotron top), as illustrated in Figure 2a. After completely filling up the rhizotrons, 100 ml of tap water was added to moisten the medium and allow initial solubilization of the fertilizers.

Soybean seeds (*G. max* L., Eiko cultivar; Asgrow, United States) were pre-germinated in Petri dishes with moistened filter paper (Herzel et al., 2020). The Petri dishes were sealed and covered with aluminum foil and kept incubated for 48 h in the greenhouse. Seedlings with equal radical sizes were then selected and transplanted, using one seedling per rhizotron (Herzel et al., 2020). The seedlings were placed in a centralized position close to the transparent plate of the rhizotrons, at a depth of approximately 2 cm from the substrate surface (Robles-Aguilar et al., 2020a). The rhizotrons were kept at a 45° inclination in a fixed randomized position, with the transparent plates facing downward, covered by black plastic sheets (Nagel et al., 2012; Robles-Aguilar et al., 2020a), as shown in Figure 2b.

The growth medium was moistened throughout the experiment with a 100–250 ml water supply 2 times per week, maintaining approximately 14–30% of the substrate field capacity. All plants were treated against downy mildew contamination with Ortiva^®^ (Syngenta, Germany), applied at 19 days from sowing. Images of the visible root system were recorded 2–3 times a week, along with measurements of the number of leaves and plant height. Harvest was conducted after 40 days of cultivation in the rhizotrons. Prior to shoot harvest, soil plant analysis development (SPAD) values were measured from trifoliate leaves at the uppermost node with a Chlorophyll Meter SPAD-502Plus (Konica Minolta). The growth medium and the roots were collected in layers, cut as illustrated in Figure 2c: A (top layer, between 0 and 10 cm depth), B (middle layer, between 10 and 30 cm depth), and C (bottom layer, below 30 cm depth). Roots were separated from the substrate samples with a sieve (9 mm × 5 mm mesh holes).

### Post-harvest Analysis

After harvesting, leaf area was determined with a leaf area meter (LI-3100, LI-COR) and, subsequently, the shoots were dried in an oven at 60°C until constant weight to determine total dry biomass. Roots were immediately stored in flasks containing 50% v/v ethanol solution and kept in a dark cooling chamber at 4°C until further analysis. Roots were carefully washed and scanned (Epson Expression 10000 XL) for measurements of total root length, average root diameter, and root surface area, using WinRHIZO Pro V 2020a software, followed by drying in the same conditions as the shoots. Dry biomass of shoots and roots were measured, and shoot:root-ratio based on biomass was calculated.

Chemical analysis of the ground biomass was determined by inductively coupled plasma optical emission spectrometry (ICP-OES; Thermo Scientific iCAP6500) for P, S, Mg, and K, and *via* CHN elemental analysis (Leco TCH 600) for N. Based on the elemental analysis results, N:S ratio was calculated. Sulfur and phosphorus use efficiency (SUE and PUE, respectively) were estimated using the following equations (Chowdhury et al., 2020):


(1) $$Uptake\left(g/pot\right)=ShootBiomass\left(g/pot\right)\times \frac{NutrientConcentration\left(\%\right)}{100}$$

(2) $$SUE\left(\%\right)=\frac{Suptake\left(fertilized\right)-Suptake\left(control\right)\left(g/pot\right)}{Sapplied\left(g/pot\right)}\times 100$$

(3) $$PUE\left(\%\right)=\frac{Puptake\left(fertilized\right)-Puptake\left(control\right)\left(g/pot\right)}{Papplied\left(g/pot\right)}\times 100$$

Homogenized substrate samples from each layer were analyzed to determine nutrient concentrations. Available S (in sulfate form) was extracted with mono-calcium phosphate and the concentration was determined turbidimetrically with a UV-Vis spectrophotometer (Femto 600plus) (Raij et al., 2001). Available P (phosphate in soil solution) was extracted with water and anionic resin, as proposed by Quaggio and Raij, and quantified using a UV-Vis spectrophotometer (Femto 600plus) (van Raij et al., 1986). Mg was extracted using a cationic resin and estimated with atomic absorption spectrophotometer (Perkin Elmer 2380). Nitrogen (total) was determined by CHN elemental analysis with a Perkin Elmer 2400 analyzer.

### Rhizotron Image Analysis

Rhizotron images were analyzed using the software *GrowScreen-Root*, according to Nagel et al. (2012). The roots were manually marked as primary roots or as first and second order lateral roots, labeled in green, red, or blue, respectively (Supplementary Figures 2–7). The length of each root type, total root length, root length density, root system depth (representing the maximal vertical distribution of a root system), and convex hull area (representing the surface area of a rhizotron covered by the whole root system) were determined.

### Statistical Analysis

All results were submitted to one-way statistical analysis (ANOVA) with Tukey’s test at the significance level *p* < 0.05 (Origin Pro 9.0, United States).

## Results and Discussion

Fertilizer composites with a controlled-release dynamic were obtained as sustainable alternatives to P and S fertilization, consisting of a polysulfide matrix (PS) as support to dispersed struvite particles (St). The fertilizers were produced with different contents of each component, namely, 25, 50, and 75 wt% of the phosphate source. The different mass ratios were studied because the synergism and interactions between struvite particles and the polysulfide matrix may differ–e.g., the dispersion and barrier effects of the matrix on struvite dissolution and release may balance one another; S oxidation can be improved with higher P amounts and P solubilization can increase with higher polysulfide oxidation into sulfate. Therefore, we wanted to test different matrix-to-P configurations to observe if they could produce different outcomes regarding P release and S oxidation.

The same materials were studied in previous work from our group, displaying a controlled-release behavior for phosphate in citric acid solution and a synergistic dynamic between S and P in soil (Valle et al., 2021). Sulfur is partially polymerized in the composite, with a fraction remaining unreacted as re-crystallized elemental sulfur (S_8_) (Valle et al., 2019, 2021). Nevertheless, the achieved polysulfide formation sufficiently provides functionality to the material, as an easily processible matrix to support struvite. Chemical characterizations of the materials in Valle et al. (2021) also revealed that, during the preparation of the composites, the struvite crystalline phase is converted to dittmarite [Mg(NH_4_)(PO_4_)⋅H_2_O], losing structural water. This phase transition does not significantly impact the fertilizer’s properties and, most importantly, it does not reduce efficiency. Dittmarite has a similar P release profile to struvite, as it tends to rapidly re-hydrate when in solution, returning to the struvite crystalline phase (Massey et al., 2009). Dittmarite is more thermally stable than struvite, which could be favorable for processing purposes (Farhana, 2015). Moreover, dittmarite presents a higher nutrient concentration, which is more interesting for agronomic purposes.

### Effect of Different Treatments on Soybean Development and Root System Architecture

Soybean (*G. max* L.) was cultivated in rhizotrons with different sources of S and P over 40 days. This crop was selected for the study due to its high demands on both P and S nutrition (Zhao et al., 2008; Soares et al., 2016; Ibañez et al., 2020). A substrate with low to moderate concentrations of P and S was used to favor the absorption of nutrients supplied *via* the fertilizers. It is worth mentioning that no phytotoxicity or micronutrient deficiency symptoms were observed over the course of the experiment. Plants grown with no additional fertilizer (control treatment) remained relatively small and did not evolve significantly over time, unlike the fertilized treatments (Supplementary Figure 1). It was possible to observe a rapid development after around 30 days of plant growth for TSP/AS, St/S8, and the St/PS composites, corresponding to the appearance of flowers (Figure 2d). As the reproductive stage starts, soybean tends to rapidly accumulate biomass to complete the vegetative development (McWilliams et al., 1999).

**FIGURE 3:**
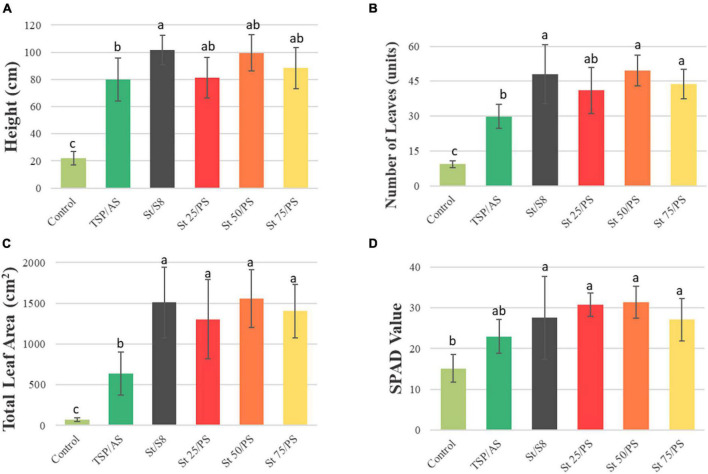
Average plant **(A)** height, **(B)** number of leaves, **(C)** total leaf area, and **(D)** SPAD value, measured before harvest, 40 days after sowing. Bars show mean values ± standard deviations. Indexes a, b, and c indicate significant differences between treatments (*p* < 0.05).

On the harvest day, measurements were carried out for the final plant height, the number of leaves, total leaf area, and SPAD values (Figure 3). Plants under the unfertilized control achieved a significantly lower performance than the others in all measurements. It is interesting to notice that the treatments containing struvite (with S_8_ or PS) were statistically superior to the positive control (TSP/AS), reaching more than double the leaf area, for instance. While TSP/AS featured on average 30 leaves per plant, St/S8 and St 50/PS displayed nearly 50 leaves. The SPAD values, which estimate the chlorophyll content of leaves, were less divergent among fertilized treatments, as expected by their development. The results indicate an increased development of soybean in the presence of struvite, demonstrating that phosphate can be efficiently provided to plants in this form. The results might also be related to the co-management of struvite with sulfur (in S^0^ oxidation state) or to the additional Mg supply. Moreover, the relatively higher application of NH_4_NO_3_ with water-soluble sources in TSP/AS probably elevated soil salinity, which is limiting to plant growth. The ammonium sulfate and nitrate sources have a saline index of 69 and 105%, respectively (Vitti et al., 1994). The rapid dissolution of these sources can increase the electrolyte concentration of the fertilized soil solution close to the roots. This high concentration of electrolytes near the seeds or roots can reduce or inhibit water absorption due to the increased osmotic pressure of the solution (Taiz et al., 2015).

**FIGURE 4:**
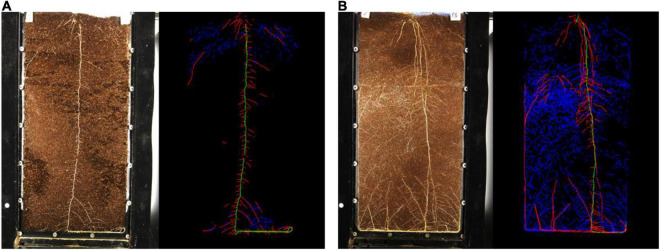
Original and analyzed color coded rhizotron images of **(A)** control with no fertilizer and **(B)** St 50/PS treatment, 40 days after sowing. Primary roots and first and second order lateral roots are represented by the colors green, red, and blue, respectively.

The root system architecture of unfertilized control plants strongly differed from the fertilized treatments, which presented pronounced second order lateral root development (Figure 4). Representative rhizotron images of all treatments over time can be found in SI (Supplementary Figures 2–7). Plants that showed greater vegetative development (i.e., struvite-based treatments) also featured a greater presence of thinner roots and a more homogeneous distribution throughout the substrate volume. It is known that lateral roots contribute the most to the absorption of water and nutrients by plants, due to their activity and capillarity in soil.

Visible root measurements from plants at 40 days of cultivation can be found in Supplementary Table 3. While the final primary root length was similar among treatments, lateral root development was more affected by the fertilizer source. St 50/PS featured the largest first and second order lateral roots, with, respectively, 565 cm and 1400 cm, which were significantly superior to TSP/AS (368 and 549 cm, respectively) and the unfertilized control (203 and 202, respectively). Moreover, struvite treatments achieved in general higher total root length than TSP/AS and control.

Plant response to nutrient availability or deficiency can be indicated by the differences in growth and in the spatial distribution of roots within the soil. In some plants, like common wall cress (*Arabidopsis thaliana*) and alfalfa (*Medicago sativa*), S deficiency has relatively little effect on root morphology and affects more negatively shoot biomass production, decreasing shoot:root ratio (Wang et al., 2003; Gruber et al., 2013). Nevertheless, soybean plants treated with S_8_ in Zhao et al. (2008) displayed an increase in lateral roots compared to a control with no S supply. Phosphorus effect on root system architecture patterns is often more species-dependent. Gruber et al. (2013) reported that *A. thaliana* plants present shallower and branched root systems under insufficient P, for instance. According to López-Bucio et al. (2003), their root system senses and responds to P deprivation locally. Lyu et al. (2016) verified that P deficiency caused a more significant decrease in root length and increase of secondary lateral roots in fibrous root species (e.g., *Zea mays*) than in legumes (e.g., *G. max*). Robles-Aguilar et al. (2020b) found that lupine (*Lupinus angustifolius* L.), a leguminous plant like soybean, increased primary root elongation in unfertilized treatments, compared to struvite fertilization. On the other hand, in a study on soybean cultivation by Milton et al. (1991), P supply promoted an increase in total root length. In Watt and Evans (2003), soybean produced more branched roots with P addition, which grew more concentrated around the area where the fertilizer was applied. In contrast, Li et al. (2017) found that soybean root length density was smaller with higher P rates.

**FIGURE 5:**
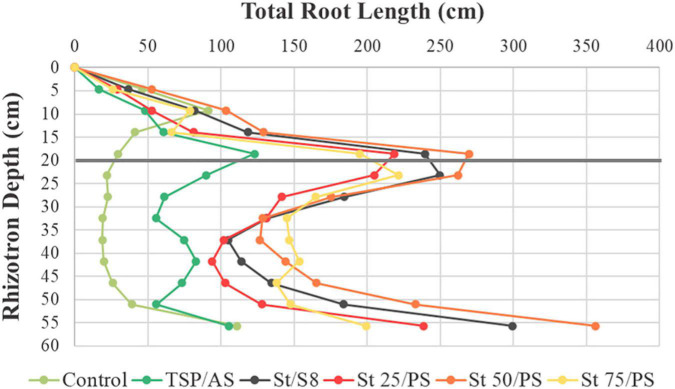
Effect of treatments on visible total root length. Trends of root length density over the rhizotron depth are shown at harvest time point (40 days after sowing). The applied fertilizer layer is at a depth of 20 cm from top (marked with the gray line). Dots represent mean values.

Figure 5 illustrates the visible root length density profiles, indicating quite some variation in spatial root distributions across the different fertilizer treatments. Pronounced root development can be found in the region around the fertilizer layer (at 20 cm from the top), except for the unfertilized control, highlighting the relation between root growth and the presence of nutrients, also noticed by Watt and Evans (2003). It should be noted that all treatments displayed an increased root length in the lowest 10 cm of the rhizotrons. Roots started to reach the bottom of the rhizotrons 10 days after sowing and, thereafter, an enhanced root development could be found along the bottom part of the rhizotrons as a consequence of the experimental design.

The lowest root length density is observed in the unfertilized control, compatible with its inferior shoot development. Unlike other treatments, the control presents a relatively larger root production closer to the substrate surface, which might be a response to P deficiency, as reported for *A. thaliana* plants (Gruber et al., 2013). Struvite-based treatments achieved a higher apparent root accumulation than TSP/AS over the rhizotron volume, especially composite St 50/PS. While the results clearly differed between struvite and TSP, plant behavior did not vary between S_8_ and PS, indicating that soybean root distribution might be more strongly related to P supply than to differences between the S^0^ sources.

**FIGURE 6:**
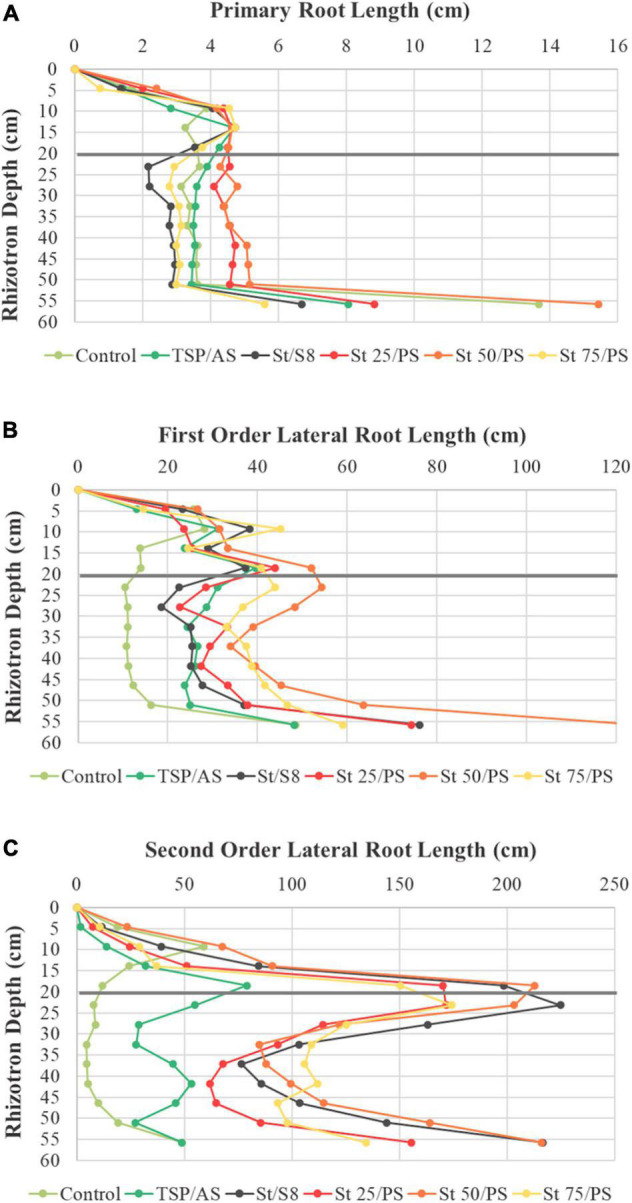
Effect of treatments on different root types: **(A)** primary roots and **(B)** first and **(C)** second order lateral roots. Trends of root length density over the rhizotron depth are shown at harvest time point (40 days after sowing). The applied fertilizer layer is at a depth of 20 cm from top (marked with the gray line). Dots represent mean values.

Root production around the fertilizer layer corresponded mainly to second-order lateral roots, as can be seen in Figure 6. The primary root growth pattern was similar in all treatments, contributing less to the total root length density results (Figure 6A). First-order lateral roots showed a maximum around the fertilizer layer and a smaller peak of accumulation in the upper layer, probably from plant anchoring (Figure 6B). Second order lateral roots occupied the largest volume of the rhizotron and could be found mainly in the fertilized region (Figure 6C). The profiles were consistent with the data found in Supplementary Table 3, with a superior second order lateral root production in struvite-treated plants than TSP/AS.

Watt and Evans (2003) correlated soybean’s high development of thinner branched roots to plant P uptake. The continuous root growth across the soil volume allows the interception of labile P from soil solution before it becomes soil-bound. The different outcomes from TSP and struvite treatments could be related to their distinct phosphate release profiles. TSP has a fast initial release of P and, therefore, phosphate was probably highly available during the first days of soybean cultivation, before undergoing immobilization processes in the substrate. In contrast, struvite is a slow-release fertilizer with an ongoing dissolution. Phosphate from struvite treatments is delivered more steadily and may be accessed by roots over a longer period of time. The increased development of thinner lateral roots in struvite treatments, highly concentrated around the fertilizer layer, are strong indications that roots continued to grow and occupy the rhizotron as a response to phosphate prolonged delivery, especially in the case of the composites.

It is interesting to notice that St/S8 had a comparable second-order lateral root length to St 50/PS, but its first order lateral root was inferior to all polysulfide treatments (Supplementary Table 3 and Figure 6). This could be related to the differences in S structure. Zhao et al. (2008) showed that S supply to soybean as S_8_ not only increased lateral root development but also the number of soil microorganisms and enzyme activity. Both PS and S_8_ require biological activity to be oxidized to sulfate, and roots may contribute to this by releasing organic compounds that stimulate soil microorganisms (van Veelen et al., 2020). Therefore, even though P supply appeared to contribute more significantly to soybean root system distribution, the S sources probably played a role in root traits as well.

The dynamic trend of root development over time revealed an increased rate of second order lateral root growth after 30 days of cultivation (Supplementary Figure 8). This result goes along with the enhanced plant height and number of leaves at the same period of time (Figure 3), corresponding to the soybean reproductive period. Trends in root system depth and convex hull area can be found in Supplementary Figure 9.

Since rhizotron images only provide information regarding visible roots, the complete root systems were measured after harvest by washing and scanning the roots (Table 1). It should be noted that the data corresponds mostly to primary and first order lateral roots. The sampling method was not adequate to collect thinner roots, as a considerable portion of the second order lateral roots was not separated from the soil during sieving, hence not contributing to the root measurements. Following the same trend from rhizotron images, St 50/PS achieved the largest total root length (4291 cm) and root surface area (593 cm^2^, Table 1). The lowest values, however, were from TSP/AS, instead of unfertilized control plants, which could be attributed to the loss of second order lateral roots, more prominent in the fertilized treatments (Supplementary Table 3).

**TABLE 1:** Effect of treatment on average total root length, root diameter, and surface area.

	Root measurements			
Treatment	Total length (cm)	Diameter (mm)	Surface area (cm^2^)
Control	1592.2 *ab*	0.34 *b*	167.0 *b*
TSP/AS	982.2 *b*	0.42 *ab*	118.3 *b*
St/S8	1571.9 *ab*	0.50 *a*	215.4 *ab*
St 25/PS	1942.0 *ab*	0.48 *a*	256.1 *ab*
St 50/PS	4290.6 *a*	0.49 *a*	592.6 *a*
St 75/PS	3674.8 *ab*	0.48 *a*	481.5 *ab*

*Indexes a and b signal significant differences between treatments (p < 0.05).*

Control plants with no fertilizer displayed a smaller average root diameter than struvite treatments (Table 1), which goes along with the reduced root and shoot development and biomass accumulation. Root diameter was also analyzed in the three different layers (Supplementary Table 4). The average root diameter of unfertilized control plants was constant in all layers (in the range of 0.33–0.35 mm). In contrast to the control, plants grown in fertilized treatments produced thicker roots in the top layer (top layer: 0.58–0.72 mm vs. bottom layer: 0.34–0.38 mm), possibly to support the higher biomass production. Plants under all treatments exhibited the highest proportion of roots in the root diameter class 0.2 and 0.3 mm (Supplementary Table 5; around 30% of the total root length). In addition, plants treated with struvite had a high proportion of thicker roots (>0.5 mm) which is less pronounced in control plants, reflecting the average results from Table 1. Nevertheless, thinner roots could be underestimated, especially in struvite treatments, which had a high second order lateral root development.

**FIGURE 7:**
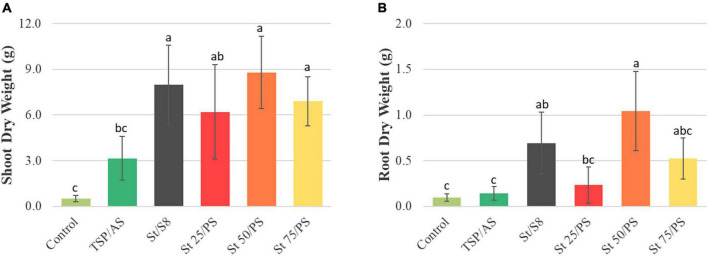
Effect of treatments on biomass from **(A)** shoots and **(B)** roots. Bars show mean values ± standard deviations. Indexes a, b, and c indicate significant differences between treatments (*p* < 0.05).

Dry biomass was measured both for shoots and roots (Figure 7). Shoot biomass was higher in treatments with struvite and significantly lower in the unfertilized control. Regarding root biomass, both plants under no fertilizer and TSP/AS treatments achieved inferior results. Plants treated with St 50/PS reached 10 times the root dry matter of TSP/AS grown plants, for instance. The fertilized treatments had comparable shoot:root ratios, superior to the unfertilized plants (Supplementary Figure 10). The relation shows that plant biomass production was predominantly directed to shoot development when additional nutrients were supplied, indicating that struvite and polysulfide were able to properly provide P and S.

Soybean cultivation with the struvite-polysulfide composites not only displayed a significant biomass production, superior to the treatment with TSP and ammonium sulfate, but also a larger root proliferation. The intense root growth could be a response to the prolonged availability of phosphate due to struvite’s slow-release character. Enhanced root growth can significantly benefit crop production, improving soil microstructure, soil porosity, and bulk density, among an overall enrichment of organic carbon in the soil. Most importantly, it implicates an increased soil rhizosphere, with a more diverse microbial community and better nutrient mobility and bioavailability. In field conditions, this is especially favorable, thereby benefiting the following crop cultivations.

### Nutrient Availability and Uptake

For a more accurate understanding of the relationship between plant development and the fertilizers, it is essential to determine the nutrient recovery, as well as P and S final concentrations in the substrate. The control plants with no fertilizer displayed a lower relative concentration of all elements in shoots compared to the other treatments, except for sulfur (Supplementary Table 6). Sulfur uptake by control plants was probably obtained from mineralization of organic S, promoted by enhanced root growth (van Veelen et al., 2020). S plays a central role in the synthesis of proteins in plants, and also in symbiotic N_2_ fixation, a process that soybean uses to assimilate nitrogen when this nutrient is deficient in soil (Becana et al., 2018). However, nodule formation on roots was not observed, suggesting that the unfertilized control plants did not fixate nitrogen. In addition, N uptake achieved by the control plant was critically low (0.74 wt%, Supplementary Table 6), possibly due to the low availability of N and other essential nutrients (IFA et al., 2016; Robles-Aguilar et al., 2020a). Furthermore, the results indicate P deficiency in the unfertilized treatment (Supplementary Table 6). Triple superphosphate provided the highest relative P concentration in shoots (1.15 wt%), although it did not outperform the other fertilized treatments for other elements. Root elemental analysis of the complete root system and from the three rhizotron layers can be found in the SI (Supplementary Tables 6, 7).

All fertilized treatments resulted in adequate N:S ratios (Table 2), essential for protein synthesis and for crop yields (Ibañez et al., 2020). The control plants with no fertilizer presented a low N:S relation due to insufficient nitrogen uptake. The highest sulfur use efficiency (SUE) was achieved by St 50/PS (22%), while the lowest efficiency was from the soluble form TSP/AS (8%). Furthermore, the triple superphosphate treatment featured the lowest phosphorus use efficiency (PUE), although at *p* < 0.05 it was comparable to the other treatments. The results indicate an efficient S oxidation from the polysulfide and sufficient struvite solubilization.

**TABLE 2:** Nutrient uptake efficiency parameters from plant biomass: average N:S ratio, sulfur use efficiency (SUE, %), and phosphorus use efficiency (PUE, %). Nutrient concentration in the substrate after soybean harvest: available phosphate (mg/dm^3^), available sulfate (mg/dm^3^), total nitrogen (mg/dm^3^), and magnesium (mg/dm^3^).

	Nutrient uptake efficiency			Nutrient concentration in soil			
Treatment	N:S	SUE (%)	PUE (%)	P available (mg/dm^3^)	S available (mg/dm^3^)	N total (mg/dm^3^)	Mg (mg/dm^3^)
Control	2.2 *b*	-	-	16.5 *b*	14.3 *d*	2790.4 *a*	211.5 *bc*
TSP/AS	15.5 *a*	8.1 *b*	10.7 *a*	74.5 *a*	53.1 *a*	3949.5 *a*	177.9 *c*
St/S8	16.2 *a*	16.0 *ab*	11.4 *a*	95.7 *a*	37.4 *c*	3647.7 *a*	255.7 *a*
St 25/PS	15.2 *a*	11.8 *b*	11.5 *a*	85.5 *a*	39.4 *bc*	3128.7 *a*	232.0 *ab*
St 50/PS	15.8 *a*	22.0 *a*	14.1 *a*	93.9 *a*	51.3 *a*	2588.9 *a*	214.3 *bc*
St 75/PS	16.2 *a*	16.2 *ab*	13.6 *a*	86.4 *a*	47.7 *ab*	3125.9 *a*	241.8 *ab*

*Indexes a, b, c, and d indicate significant differences between treatments (p < 0.05).*

The concentration of available phosphate in the rhizotron was statistically similar between the different fertilized treatments, ranging from 75 to 96 mg/dm^3^ (Table 2). Considering that TSP/AS is readily soluble, this result indicates the immobilization or loss of P from this source, reducing the expected fertilizer efficiency. Struvite treatments, on the other hand, have a controlled-release behavior and may have not fully solubilized up to that point. In a long-term assessment with ryegrass, Bogdan et al. (2021) found that significant struvite dissolution and phosphate release were only observed after 4 months of cultivation.

In the unfertilized control, available P presented no distinction between the three soil layers (Supplementary Table 8). This shows that phosphate mobilization from the substrate by root exudates occurred equally over the rhizotron profile, as root length was relatively similar in all layers of the unfertilized control. In contrast, the middle layer (B) from TSP/AS and struvite treatments featured a significantly higher available P concentration, ranging from 164 to 237 mg/dm^3^, while values from the top and bottom layers (A and C) were closer to the unfertilized control (around 20 mg/dm^3^). This result shows the typical low mobility and diffusion of phosphate, observed in agricultural soils in general. Furthermore, it is consistent with the assumption that root proliferation in the middle layer (Figures 5, 6) was associated with struvite ongoing dissolution.

The highest available sulfur concentration in the substrate was from TSP/AS and St 50/PS, while St/S8 achieved the lowest (Table 2). Since phosphate presence tends to block soil SO_4_^2–^ adsorption sites, this explains why sulfate from the soluble source (AS) remains highly available (Scherer, 2001). The results also reveal that S oxidation into sulfate was more effective from PS in the composites than from S_8_, which is compatible with the hypothesis that S_8_ and PS different S forms could have altered effects on the substrate microbial activity and plant growth dynamics. Sulfate concentration in the unfertilized control indicates S mineralization by root exudates, as discussed in the shoot recovery results. Contrary to phosphate, the middle and bottom layers have similar soil S contents (Supplementary Table 8), indicating sulfate had better transportation over the substrate depth.

High *N* values in the substrate reveal a low incidence of N volatilization and high organic N content (Table 2). St/S_8_ treatment achieved a superior Mg concentration in the substrate by the end, which was expected from struvite composition. The other treatments displayed significant Mg concentrations, including the unfertilized control and TSP/AS, indicating a great mobilization from the organic fraction of the substrate. Moreover, this suggests Mg content in struvite was not decisive for the better performance and vegetative development of St/S_8_ and St/PS treatments. Based on these results, the lower Mg and N uptake by the unfertilized control plant was mostly related to insufficient P on the substrate.

## Conclusion

The elucidation of plant–soil dynamics and roots growth patterns under struvite-polysulfide fertilization is important to understand and validate the agronomic efficiency of this new class of slow-release fertilizers. Hence, sustainable fertilizers with a polysulfide matrix and dispersed struvite (containing 25, 50, or 75 wt% of struvite) were prepared, using the simple and green method of inverse vulcanization. The effect of P and S supply from this system on soybean cultivation was compared both to the co-management of soluble commercial sources [TSP and (NH_4_)_2_SO_4_] and to pure struvite mixed with S_8_. The results revealed a significantly higher biomass production from the combined application of struvite with S^0^ sources (polysulfide or S_8_) than with the TSP/(NH_4_)_2_SO_4_ treatment. Struvite achieved a similar phosphorus use efficiency as the TSP reference, proving its controlled-release behavior can properly provide P to plants in the studied conditions. The composite St 50/PS displayed the greatest sulfur use efficiency, superior to the fine particles from S_8_ powder and to ammonium sulfate, which reached the lowest SUE. Root system architecture analysis using rhizotrons revealed an intense accumulation of second order lateral roots around the fertilizer layer, especially in struvite treatments. The higher development of thinner roots was attributed to the slow-release and continuous availability of phosphate from struvite, in contrast to TSP quick solubilization and P losses. Although root traits were more significantly influenced by the P source, differences in first order lateral root lengths from PS and S_8_ treatments could be related to the S structure and its influence on the local microbial activity. The final concentration of sulfate in the growth medium also indicated superior oxidation of S from the polysulfide than S_8_. In summary, the slow-release struvite-polysulfide composites proved to be efficient fertilizer alternatives to soluble commercial sources, and beneficial to soybean development.

## Data Availability Statement

The original contributions presented in the study are included in the article/Supplementary Material, further inquiries can be directed to the corresponding authors.

## Author Contributions

SFV conceived and designed the experiments, collected the data, performed the analysis, and wrote the manuscript. ASG, GGFG, AG, KAN, NDJ, and CR conceived and designed the experiments and wrote the manuscript. JC collected the data and performed the analysis. All authors contributed to the article and approved the submitted version.

## Conflict of Interest

The authors declare that the research was conducted in the absence of any commercial or financial relationships that could be construed as a potential conflict of interest.

## Publisher’s Note

All claims expressed in this article are solely those of the authors and do not necessarily represent those of their affiliated organizations, or those of the publisher, the editors and the reviewers. Any product that may be evaluated in this article, or claim that may be made by its manufacturer, is not guaranteed or endorsed by the publisher.
