# Supplementary material for: Co-fertilization of Sulfur and Struvite-Phosphorus in a Slow-Release Fertilizer Improves Soybean Cultivation
Source: Front Plant Sci. 2022 May 10;13:861574. doi: 10.3389/fpls.2022.861574 (PMC9127873; doi:10.3389/fpls.2022.861574)
Supplement: Supplementary file 1 [file Presentation_1.pdf]

Supplementary Information

Co-fertilization of sulfur and struvite-phosphorus in a slow-release fertilizer improves soybean cultivation

Stella F. Valle [a,b], Amanda S. Giroto [b], Gelton G. F. Guimarães [c], Kerstin A. Nagel [d], Anna Galinski [d], Jens Cohnen [d], Nicolai D. Jablonowski [d], Caue Ribeiro [b]*.*

[a] Federal University of São Carlos, Department of Chemistry, Washington Luiz Highway, km 235, 13565-905, São Carlos, SP, Brazil.

[b] Embrapa Instrumentation, XV de Novembro Street, n 1452, 13560-970, São Carlos, SP, Brazil.

[c] Agricultural Research and Rural Extension Company of Santa Catarina, Antônio Heil Highway, km 6800, 88318-112, Itajaí, Santa Catarina, Brazil.

[d] Forschungszentrum Jülich GmbH, Institute of Bio- and Geosciences, IBG-2: Plant Science, 52425, Jülich, Germany.

*email: n.d.jablonowski@fz-juelich.de; caue.ribeiro@embrapa.br.

Table of Contents

Fertilizers Contents and Applied Concentrations S3

Substrate Characterization S4

Plant Growth Trends Over Time S5

Rhizotron Images S6

Rhizotron Root Measurements S12

Rhizotron Root Growth Trends Over Time S13

Root Scanning Measurements S15

Shoot:Root Ratio S16

Plant Biomass Elemental Analysis S17

Substrate Elemental Analysis by Depth Layers S19

Fertilizers Contents and Applied Concentrations

Table S1. Treatments and respective fertilizers used to complete doses of 50 mg of S/kg, 200 mg of P/kg, and 300 mg of N/kg.

Treatment	Fertilizer	%S	%P	%N	fert mg/soil kg
**Control**	-	-	-	-	-
**TSP/AS**	TSP	-	18	-	1111.1
	Ammonium sulfate	24	-	21	208.3
	NH_4NO_3	-	-	33	776.5
**St/S8**	Elemental Sulfur (S_8)	98	-	-	51.0
	Struvite	-	11.1	6.3	1801.8
	NH_4NO_3	-	-	33	565.1
**St 25/PS**	25 wt% struvite/ 75 wt% polysulfide	40.8	3.4	1.7	122.5
	Struvite	-	11.1	6.3	1764.3
	NH_4NO_3	-	-	33	566.0
**St 50/PS**	50 wt% struvite/ 50 wt% polysulfide	29.8	7.8	3	167.8
	Struvite	-	11.1	6.3	1683.9
	NH_4NO_3	-	-	33	572.4
**St 75/PS**	75 wt% struvite/ 25 wt% polysulfide	15.1	10.7	5.4	331.1
	Struvite	-	11.1	6.3	1482.6
	NH_4NO_3	-	-	33	571.9

Substrate Characterization

Table S2: Substrate characterization. Field capacity was estimated to be around 39.1%.

Substrate characterization		
**Parameter**	**Unit**	**Value**
dry substance	%	57.2
bulk density (wet)	g/dm^3	435.0
bulk density (dry)	g/dm^3	249.0
pH (in CaCl)		5.8
conductivity (in H_2O)	µS/cm	32.0
salt (as KCl in H_2O)	g/dm^3	< 0.1
salt (as KCl in $CaSO_4$)	g/dm^3	< 0.1
nitrogen (mineral)	mg/kg	36.0
ammonium-N	mg/kg	30.0
nitrate-N	mg/kg	6.0
phosphorus (P_2O_5)	mg/kg	44.0
potassium (K_2O)	mg/kg	44.0
magnesium	mg/kg	320.0
sulfur (in SO_4^{2-})	mg/kg	33.6
organic carbon	%	19.3

Plant Growth Trends Over Time

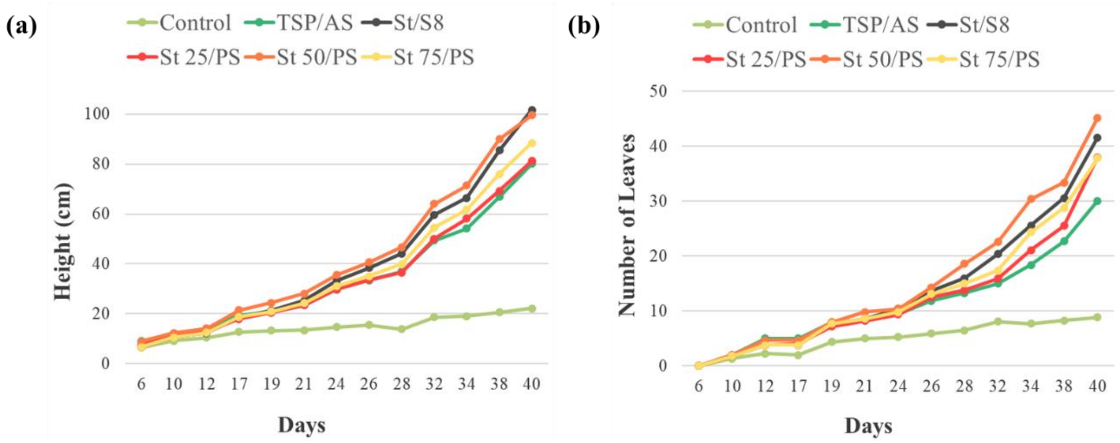

Figure S1: Dynamic growth trends of average (a) plant height and (b) number of leaves over time (n = 9 for Control and St 50/PS, n=8 for St/S8, St 25/PS, and St 75/PS, and n=7 for TSP/AS).

Rhizotron Images

Root system development and architectural changes over the time of plant growth in substrate-filled rhizotrons, **Figure S2-S7**.

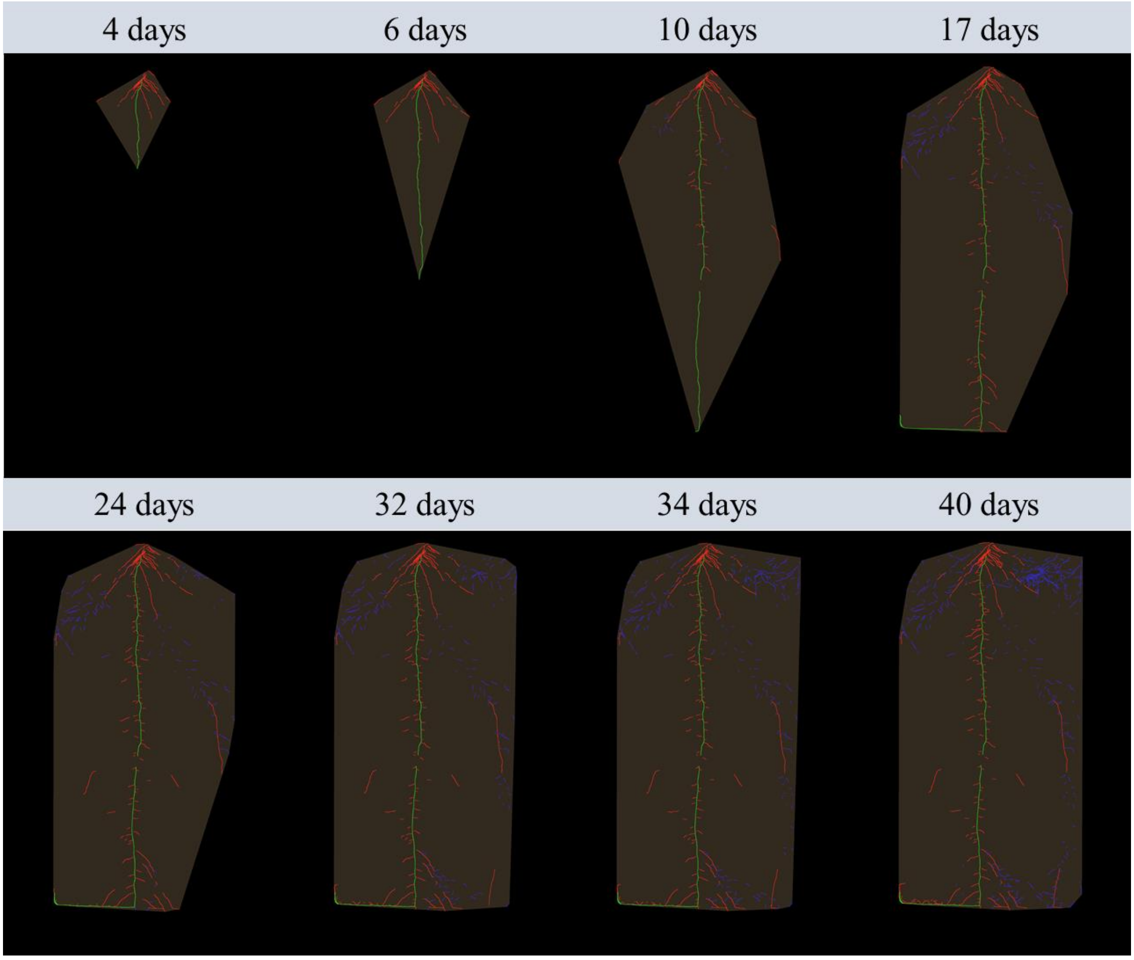

Figure S2: Color coded rhizotron images of the control with no fertilizer over time. Primary roots, first order lateral roots and second order lateral roots are represented by the colors green, red and blue, respectively.

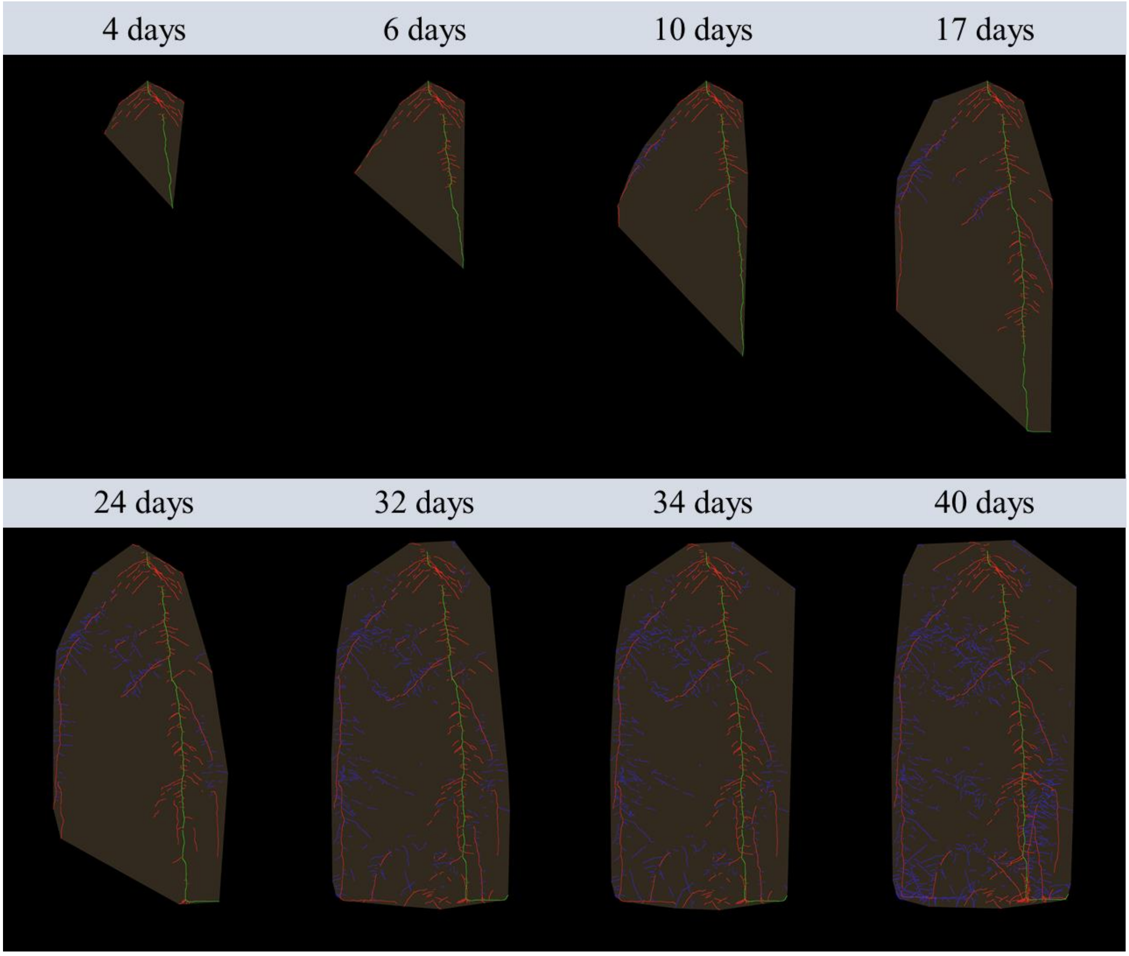

Figure S3: Color coded rhizotron images of the positive control (TSP/AS), with triple superphosphate and ammonium sulphate, over time. Primary roots, first order lateral roots and second order lateral roots are represented by the colors green, red and blue, respectively.

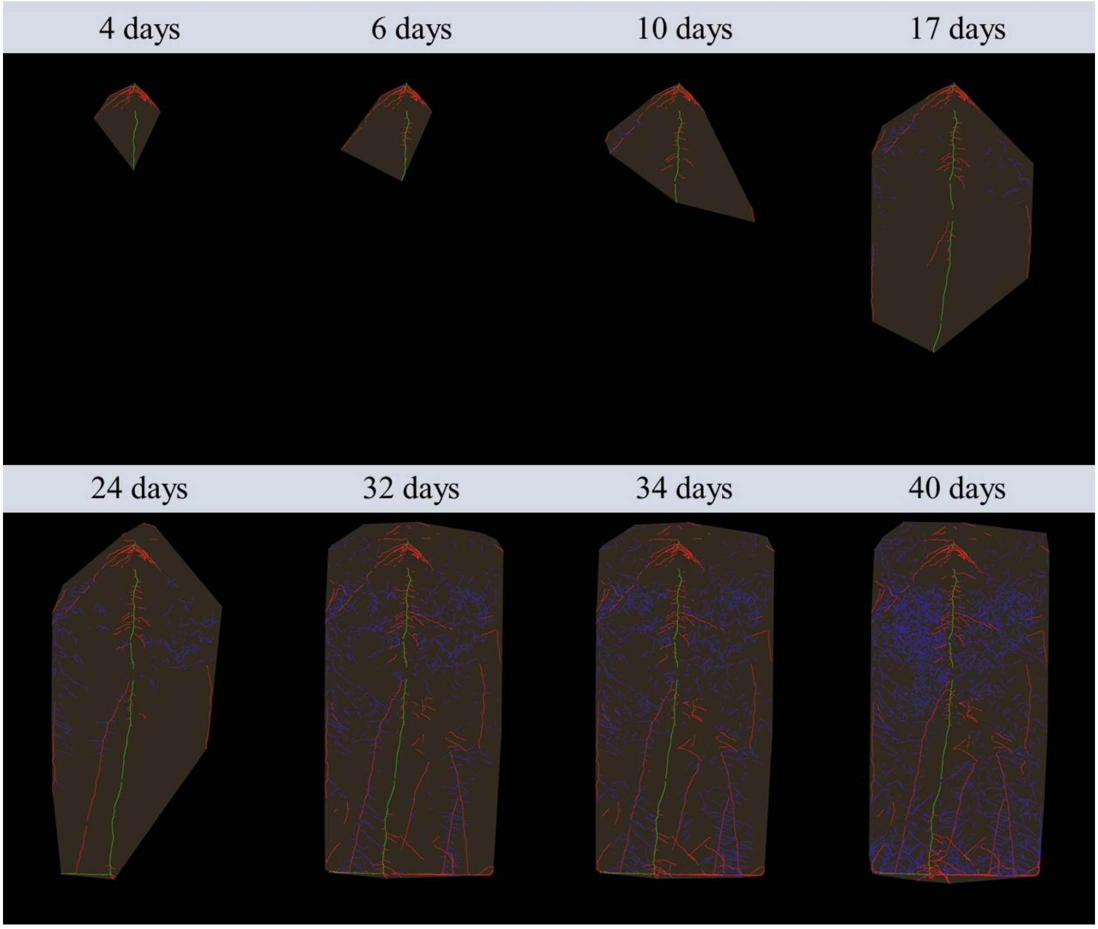

Figure S4: Color coded rhizotron images of the pure elemental sulfur and struvite mixture (St/S8) over time. Primary roots, first order lateral roots and second order lateral roots are represented by the colors green, red and blue, respectively.

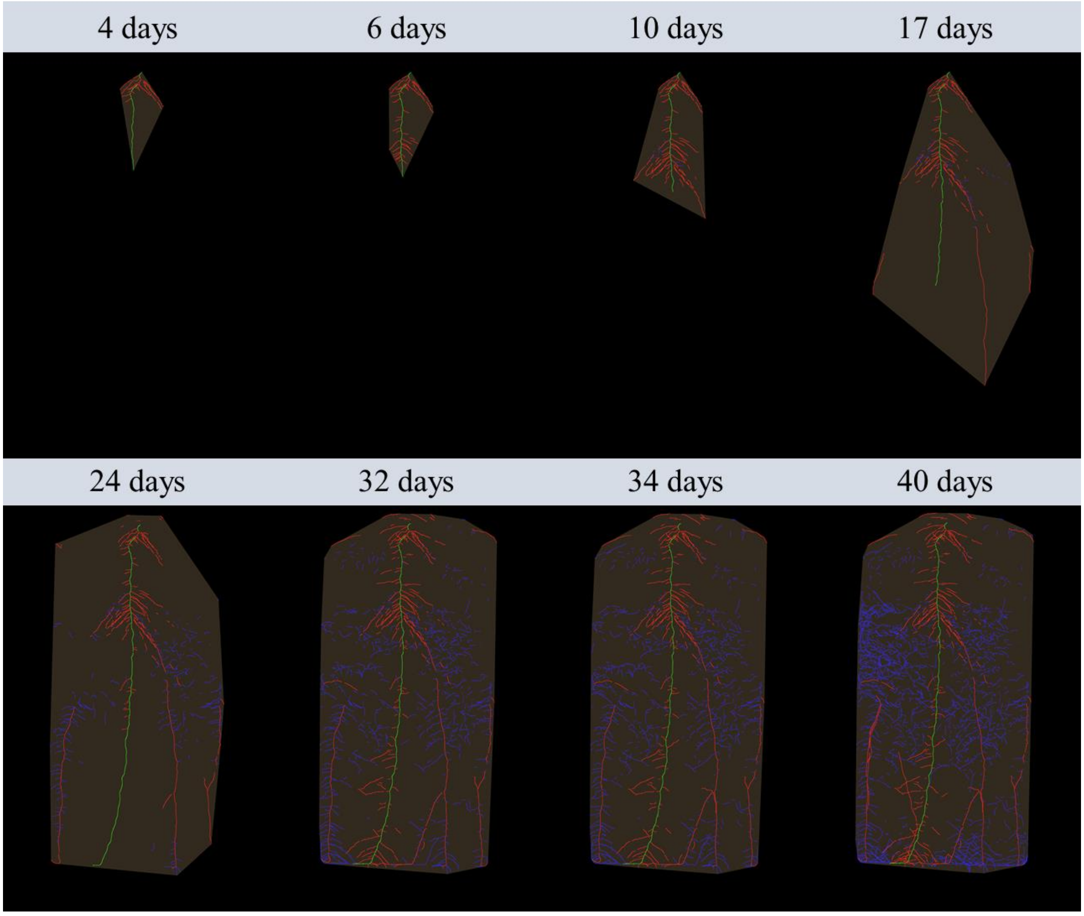

Figure S5: Color coded rhizotron images of the composite with 25 wt% struvite and 75 wt% polysulfide (St 25/PS) over time. Primary roots, first order lateral roots and second order lateral roots are represented by the colors green, red and blue, respectively.

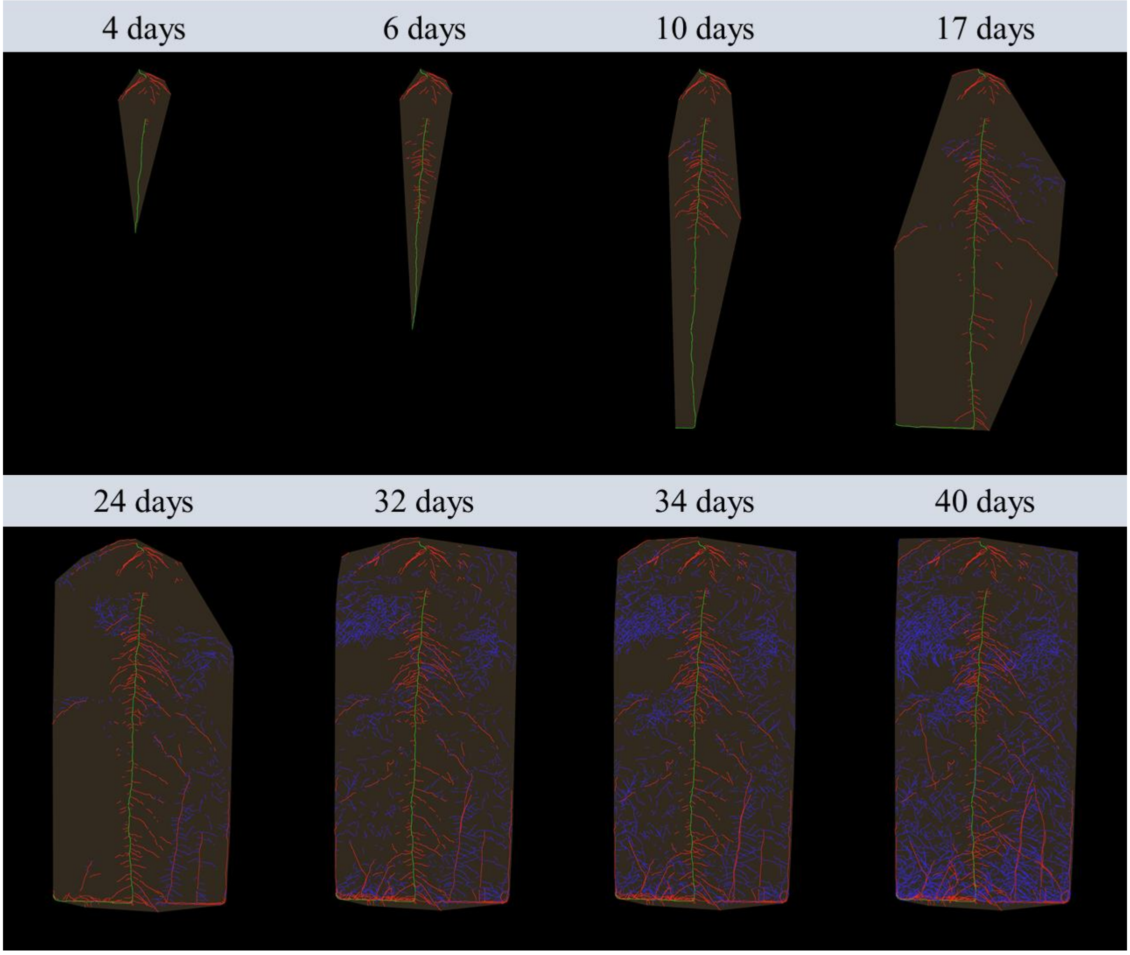

Figure S6: Color coded rhizotron images of the composite with 50 wt% struvite and 50 wt% polysulfide (St 50/PS) over time. Primary roots, first order lateral roots and second order lateral roots are represented by the colors green, red and blue, respectively.

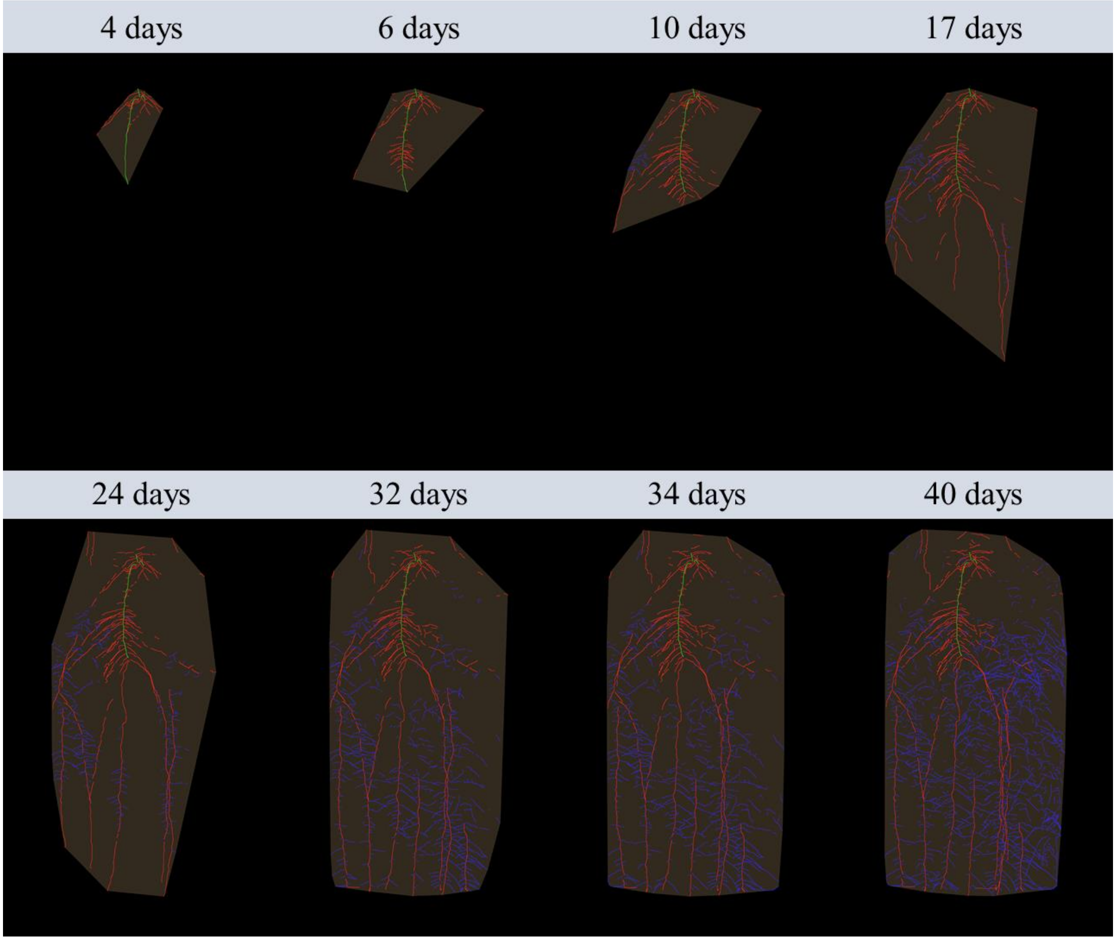

Figure S7: Color coded rhizotron images of the composite with 75 wt% struvite and 25 wt% polysulfide (St 75/PS) over time. Primary roots, first order lateral roots and second order lateral roots are represented by the colors green, red and blue, respectively.

Rhizotron Root Measurements

Table S3: Average visible root lengths (primary roots, first and second order lateral roots, and the total), root system depth and convex hull area, at 40 days after sowing. Indexes a, b, and c represent statistical differences between treatments ($p < 0.05$).

**Treatment**	Primary Root Length (cm)	First Order Lateral Root Length (cm)	Second Order Lateral Root Length (cm)
**Control**	47.3 *a*	202.5 *c*	202.2 *c*
**TSP/AS**	42.7 *a*	368.2 *bc*	548.8 *bc*
**St/S8**	41.1 *a*	357.5 *bc*	1246.5 *a*
**St 25/PS**	55.5 *a*	377.4 *bc*	910.9 *ab*
**St 50/PS**	63.1 *a*	565.1 *a*	1400.2 *a*
**St 75/PS**	40.9 *a*	430.7 *ab*	1069.6 *ab*
**Treatment**	Total Root Length (cm)	Root System Depth (cm)	Convex Hull Area (cm^2)
**Control**	452.0 *c*	52.5 *a*	1116.8 *a*
**TSP/AS**	959.8 *bc*	50.4 *a*	1155.6 *a*
**St/S8**	1645.1 *ab*	53.5 *a*	1291.4 *a*
**St 25/PS**	1343.9 *ab*	53.4 *a*	1262.7 *a*
**St 50/PS**	2028.5 *a*	53.8 *a*	1355.2 *a*
**St 75/PS**	1541.2 *ab*	52.4 *a*	1270.0 *a*

Rhizotron Root Growth Trends Over Time

Primary root length stayed relatively constant from day 17 on in rhizotrons (**Figure S8a**), as the roots reached the bottom of the rhizotrons. First order lateral roots showed a more continuous rate, and second order lateral roots presented an enhanced growth rate after 30 days (**Figure S8b,c**). This exponential growth corresponds to the beginning of the reproductive period, when roots proliferate in search of more nutrients for the intensive biomass accumulation required at this phase.

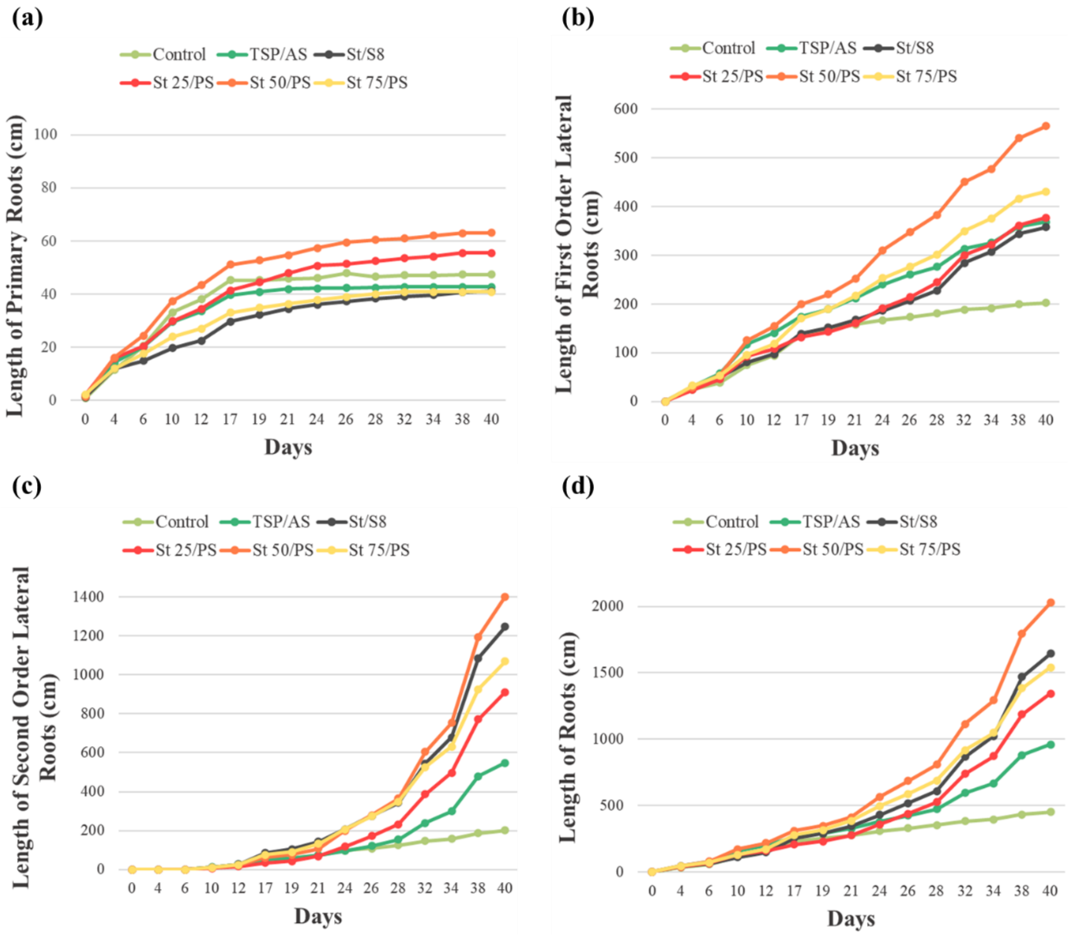

Figure S8: Dynamic growth trends of average total root length of (a) primary roots, (b) first order lateral roots, (c) second order lateral roots, and (d) their sum, over the time of plant cultivation (n = 10).

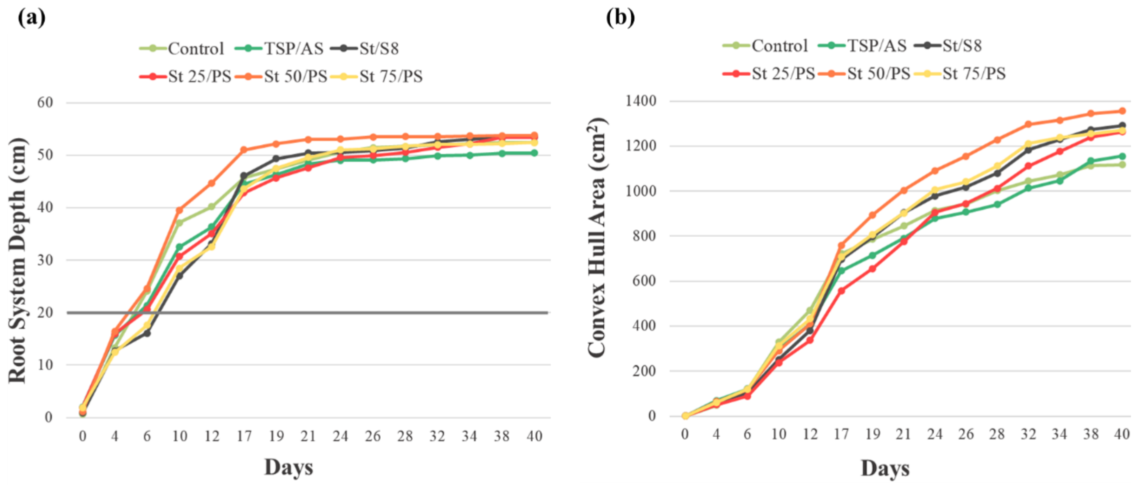

Figure S9: Dynamic growth trends of average (a) root system depth and (b) convex hull area over the time of plant cultivation (n = 10). The fertilizer layer is marked (grey line) in (a) at 20 cm from the rhizotron top surface.

Root system depth development over time (**Figure S9a**) discovered that roots from St/S8 and St 75/PS took longer (between 1 and 4 days) than the other treatments to reach the fertilizer layer. Primary root growth might be the main reason for this difference, as it was also slower under St/S8 and St 75/PS treatments compared to the other treatments (**Figure S8a**). Convex hull area (**Figure S9b**) displayed a significant increase after 17 days, which corresponds to primary roots reaching the rhizotron bottom and second order lateral roots beginning to occupy the rhizotron volume.

Root Scanning Measurements

Table S4: Average total root length, root diameter, and root surface area of each rhizotron depth layer. A= top layer (10 cm), B = middle layer (20 cm), and C = bottom layer (~26 cm). Indexes a and b signal statistical differences between layers for each treatment individually ($p < 0.05$).

Root Measurements				
**Treatment**	**Layer**	Total Length (cm)	Diameter (mm)	Surface Area (cm^2)
**Control**	A	640.8 *a*	0.35 *a*	71.8 *a*
	B	372.6 *a*	0.33 *a*	37.1 *a*
	C	578.8 *a*	0.33 *a*	58.1 *a*
**TSP/AS**	A	217.1 *a*	0.58 *a*	38.6 *a*
	B	315.6 *a*	0.35 *b*	34.1 *a*
	C	449.5 *a*	0.34 *b*	45.5 *a*
**St/S8**	A	216.0 *a*	0.72 *a*	46.1 *a*
	B	496.7 *a*	0.42 *b*	66.7 *a*
	C	859.3 *a*	0.35 *b*	102.6 *a*
**St 25/PS**	A	254.3 *a*	0.68 *a*	53.8 *a*
	B	566.3 *a*	0.42 *b*	79.2 *a*
	C	1121.4 *a*	0.35 *b*	123.0 *a*
**St 50/PS**	A	526.5 *b*	0.61 *a*	101.9 *b*
	B	1348.5 *ab*	0.46 *b*	190.1 *ab*
	C	2415.6 *a*	0.38 *b*	300.5 *a*
**St 75/PS**	A	464.7 *b*	0.66 *a*	84.9 *a*
	B	1374.1 *ab*	0.42 *b*	180.1 *a*
	C	1836.0 *a*	0.37 *b*	216.4 *a*

Table S5: Average relative root length (%) partitioned in diameter classes. Root lengths from each section were divided by the total root length (of all sections). Indexes a and b signal statistical differences between treatments, in relation to the diameter section ($p < 0.05$).

	Relative Root Length (%)					
	Diameter section (mm)					
**Treatment**	0 < D ≤ 0.1	0.1 < D ≤ 0.2	0.2 < D ≤ 0.3	0.3 < D ≤ 0.4	0.4 < D ≤ 0.5	D > 0.5
**Control**	5.7 *a*	25.7 *a*	32.8 *a*	13.4 *a*	11.1 *a*	11.2 *b*
**TSP/AS**	5.3 *a*	20.1 *ab*	27.9 *a*	15.5 *a*	14.0 *a*	17.2 *ab*
**St/S8**	4.2 *a*	16.5 *b*	27.0 *a*	15.7 *a*	12.2 *a*	24.4 *a*
**St 25/PS**	4.5 *a*	17.8 *ab*	27.7 *a*	16.6 *a*	12.8 *a*	20.7 *a*
**St 50/PS**	3.5 *a*	14.7 *b*	27.9 *a*	17.0 *a*	13.0 *a*	23.8 *a*
**St 75/PS**	4.1 *a*	18.1 *b*	29.6 *a*	15.5 *a*	12.0 *a*	20.7 *a*

Shoot:Root Ratio

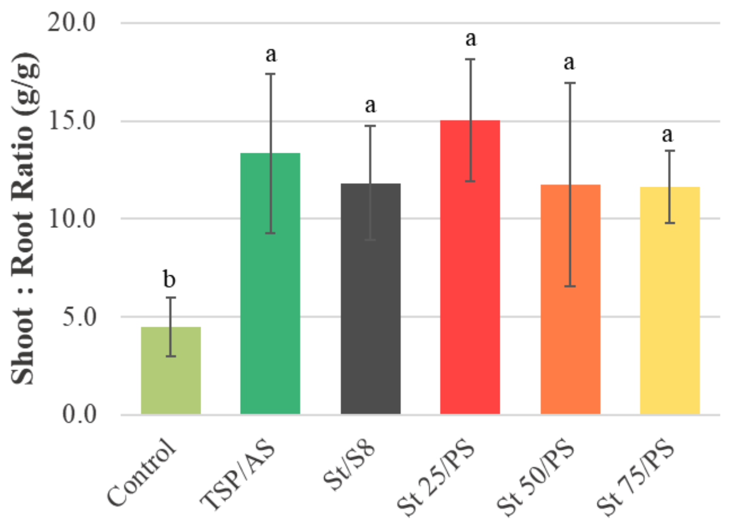

Figure S10: Effects on shoot:root ratio, related to plant dry biomass (n = 7 for Control, n=6 for St/S8, and n=5 for TSP/AS, St 25/PS, St 50/PS, and St 75/PS). Bars show mean values ± standard deviations. Indexes a and b identify significant statistical differences between treatments ($p < 0.05$).

Plant Biomass Elemental Analysis

Table S6. Average shoot (top) and root (bottom) elemental composition (wt%). Indexes a, b, and c indicate significant differences between the treatments, related to each element ($p < 0.05$).

Shoot Elemental Composition (wt %)					
**Treatment**	P	S	N	Mg	K
**Control**	0.28 *c*	0.34 *a*	0.74 *c*	0.28 *b*	1.22 *c*
**TSP/AS**	1.15 *a*	0.27 *b*	4.16 *ab*	0.36 *ab*	2.50 *a*
**St/S8**	0.78 *b*	0.23 *b*	3.70 *b*	0.35 *ab*	2.22 *ab*
**St 25/PS**	0.99 *ab*	0.26 *b*	4.01 *ab*	0.38 *a*	2.53 *ab*
**St 50/PS**	0.76 *b*	0.28 *b*	4.34 *ab*	0.44 *a*	2.02 *b*
**St 75/PS**	0.92 *ab*	0.28 *b*	4.52 *a*	0.44 *a*	2.35 *ab*

Root Elemental Composition (wt %)					
**Treatment**	P	S	N	Mg	K
**Control**	0.08 *b*	0.10 *a*	1.09 *b*	0.09 *a*	0.02 *a*
**TSP/AS**	0.17 *a*	0.09 *a*	1.96 *a*	0.08 *a*	0.05 *a*
**St/S8**	0.16 *a*	0.10 *a*	1.55 *ab*	0.09 *a*	0.06 *a*
**St 25/PS**	0.16 *a*	0.09 *a*	1.50 *ab*	0.08 *a*	0.06 *a*
**St 50/PS**	0.15 *ab*	0.10 *a*	1.58 *ab*	0.10 *a*	0.04 *a*
**St 75/PS**	0.15 *a*	0.11 *a*	1.82 *a*	0.09 *a*	0.04 *a*

Table S7: Average root elemental composition (wt %) from each depth layer. A= top layer (10 cm), B = middle layer (20 cm), and C = bottom layer (~26 cm). Indexes a, b, and c signal statistical differences between layers of each element, for each treatment individually ($p < 0.05$). Not enough material was available from Control-C and TPS/AS-B to perform the ICP-OES analysis to determine P, S, Mg, and K concentrations (indicated with a bar). Therefore, statistical analysis was not conducted to compare layers from the Control and TPS/AS regarding those elements.

Root Elemental Composition (wt %)							
**Treatment**	**Layer**	P	S	N	Mg	K	C
**Control**	A	0.08	0.10	0.8 *a*	0.08	0.02	43.93 *a*
	B	0.07	0.12	1.20 *a*	0.11	0.03	42.97 *a*
	C	-	-	1.20 *a*	-	-	43.23 *a*
**TSP/AS**	A	0.17	0.07	1.19 *b*	0.07	0.05	44.97 *a*
	B	-	-	2.36 *a*	-	-	43.86 *a*
	C	0.18	0.17	2.71 *a*	0.12	0.01	43.66 *a*
**St/S8**	A	0.14 *a*	0.06 *b*	0.86 *b*	0.07 *b*	0.09 *a*	44.69 *a*
	B	0.20 *a*	0.13 *a*	1.95 *a*	0.12 *a*	0.04 *a*	44.06 *a*
	C	0.13 *a*	0.12 *a*	2.18 *a*	0.09 *b*	0.02 *a*	43.93 *a*
**St 25/PS**	A	0.16 *a*	0.06 *b*	0.99 *b*	0.07 *a*	0.09 *a*	44.23 *a*
	B	0.16 *a*	0.12 *a*	1.85 *a*	0.09 *a*	0.04 *a*	44.24 *a*
	C	0.14 *a*	0.12 *a*	1.98 *a*	0.08 *a*	0.02 *a*	44.32 *a*
**St 50/PS**	A	0.13 *a*	0.06 *c*	0.92 *b*	0.09 *a*	0.05 *a*	45.75 *a*
	B	0.16 *a*	0.11 *b*	1.72 *a*	0.10 *a*	0.04 *ab*	44.92 *b*
	C	0.15 *a*	0.15 *a*	2.18 *a*	0.12 *a*	0.02 *b*	44.67 *b*
**St 75/PS**	A	0.14 *a*	0.07 *b*	1.06 *b*	0.07 *b*	0.05 *a*	44.72 *a*
	B	0.20 *a*	0.13 *a*	2.18 *ab*	0.10 *a*	0.03 *ab*	43.88 *b*
	C	0.15 *a*	0.16 *a*	2.44 *a*	0.12 *a*	0.02 *b*	43.65 *b*

Substrate Elemental Analysis by Depth Layers

Table S8: Substrate concentration of available P (mg/dm^3) and available S (mg/dm^3) after soybean harvest, from each depth layer. A= top layer (10 cm), B = middle layer (20 cm), and C = bottom layer (~26 cm). Indexes a, b, and c signal statistical differences between layers, for each treatment and nutrient individually ($p < 0.05$).

Treatment	Layer	P available (mg/dm^3)	S available (mg/dm^3)	N total (mg/dm3)
**Control**	A	15.8 *a*	8.0 *b*	3144.0 *a*
	B	17.7 *a*	9.7 *b*	3218.7 *a*
	C	16.0 *a*	20.2 *a*	2325.0 *a*
**TSP/AS**	A	16.8 *b*	7.8 *b*	2568.0 *b*
	B	164.0 *a*	56.0 *a*	4721.5 *a*
	C	28.8 *b*	68.8 *a*	3886.9 *ab*
**St/S8**	A	15.9 *b*	8.2 *b*	2640.0 *a*
	B	236.6 *a*	45.6 *a*	3763.0 *a*
	C	18.0 *b*	42.3 *a*	3946.5 *a*
**St 25/PS**	A	17.8 *b*	8.7 *c*	3456.0 *a*
	B	207.4 *a*	35.1 *b*	3266.0 *a*
	C	17.6 *b*	54.6 *a*	2897.3 *a*
**St 50/PS**	A	14.9 *b*	8.3 *b*	2220.0 *a*
	B	222.1 *a*	55.6 *a*	2698.0 *a*
	C	18.1 *b*	64.6 *a*	2646.9 *a*
**St 75/PS**	A	18.0 *b*	15.9 *b*	3120.0 *a*
	B	202.4 *a*	55.1 *a*	3550.0 *a*
	C	25.1 *b*	54.9 *a*	2801.9 *a*
